# Progress in the Application of Artificial Intelligence in Ultrasound-Assisted Medical Diagnosis

**DOI:** 10.3390/bioengineering12030288

**Published:** 2025-03-13

**Authors:** Li Yan, Qing Li, Kang Fu, Xiaodong Zhou, Kai Zhang

**Affiliations:** 1 Institute of Medical Research, Northwestern Polytechnical University, Xi’an 710072, China; yanli1130@nwpu.edu.cn (L.Y.); fukang@mail.nwpu.edu.cn (K.F.); 2 Ultrasound Diagnosis & Treatment Center, Xi’an International Medical Center Hospital, Xi’an 710100, China; 3 Department of Dermatology and Aesthetic Plastic Surgery, Xi’an No. 3 Hospital, The Affiliated Hospital of Northwest University, Xi’an 718000, China

**Keywords:** ultrasound, artificial intelligence (AI), deep learning (DL), machine learning (ML), diagnosis, therapy, medicine

## Abstract

The integration of artificial intelligence (AI) into ultrasound medicine has revolutionized medical imaging, enhancing diagnostic accuracy and clinical workflows. This review focuses on the applications, challenges, and future directions of AI technologies, particularly machine learning (ML) and its subset, deep learning (DL), in ultrasound diagnostics. By leveraging advanced algorithms such as convolutional neural networks (CNNs), AI has significantly improved image acquisition, quality assessment, and objective disease diagnosis. AI-driven solutions now facilitate automated image analysis, intelligent diagnostic assistance, and medical education, enabling precise lesion detection across various organs while reducing physician workload. AI’s error detection capabilities further enhance diagnostic accuracy. Looking ahead, the integration of AI with ultrasound is expected to deepen, promoting trends in standardization, personalized treatment, and intelligent healthcare, particularly in underserved areas. Despite its potential, comprehensive assessments of AI’s diagnostic accuracy and ethical implications remain limited, necessitating rigorous evaluations to ensure effectiveness in clinical practice. This review provides a systematic evaluation of AI technologies in ultrasound medicine, highlighting their transformative potential to improve global healthcare outcomes.

## 1. Introduction

The rapid advancement of medical imaging technology, coupled with the increasing volume of clinical imaging data and the growing demand for improved patient consultation efficiency and satisfaction, has rendered timely and accurate diagnosis, classification, and prognostic evaluation of diseases focal points of contemporary research. In recent years, the swift development of artificial intelligence (AI) technology has facilitated its widespread and profound application in the healthcare sector. Notably, in the field of ultrasound medicine, the introduction of key technologies such as machine learning (ML), and medical big data has revolutionized traditional diagnostic methods [1,2]. These technological advancements have led to the creation of robust, adaptable AI models that improve image acquisition and allow for real-time quality assessment. They also offer objective disease detection and diagnosis while streamlining clinical workflows within ultrasound practices [3,4].

The integration of AI in medical imaging has demonstrated modality-specific applications, each presenting unique advantages and challenges. While AI in X-ray and CT imaging primarily leverages extensive standardized image databases to detect and diagnose pathologies automatically, the static nature of these imaging modalities constrains AI’s role in real-time diagnostics [5,6]. Magnetic Resonance Imaging (MRI), with its high resolution and tissue contrast, offers rich analytical potential for AI, yet its high cost, extended scanning times, and complex image reconstruction processes have directed AI efforts toward enhancing image quality and reducing scan durations. Conversely, ultrasound, as a dynamic and real-time imaging modality, possesses distinct advantages. Its real-time capability enables AI to facilitate dynamic monitoring and guided interventions, a feature unparalleled by other imaging techniques. Additionally, the portability and cost-effectiveness of ultrasound render it particularly valuable in resource-limited settings and remote healthcare. However, unlike X-ray and CT, ultrasound images are more subject to operator variability and factors such as probe selection and tissue matching, which significantly impact image quality [7]. These factors impose higher demands on the robustness and adaptability of AI systems in ultrasound applications. Consequently, AI-driven ultrasound diagnostics not only holds the promise of enhancing diagnostic accuracy and efficiency but also offers distinctive benefits in clinical scenarios requiring rapid, cost-effective, and flexible diagnostic solutions.

Ultrasound, as a non-invasive, convenient, and cost-effective diagnostic and therapeutic modality, effectively reflects the morphology and function of human tissues and organs, thereby playing a significant role in clinical applications. However, in actual ultrasound diagnostic practice, results may exhibit deviations influenced by the physician’s experience and subjective factors [8]. Furthermore, the high workload associated with ultrasound examinations and the relatively low efficiency of some practitioners indicate considerable room for improvement in patient satisfaction with ultrasound diagnostics and treatment.

The deep integration of AI with ultrasound technology has not only enhanced the accuracy and efficiency of diagnostics but has also propelled the intelligent advancement of ultrasound medical technology. This review is organized as follows: Section 1 delineates the technological foundations of AI in ultrasound medicine, encompassing machine learning, deep learning, and convolutional neural networks. Section 2 systematically reviews current AI applications in ultrasound diagnostics, including assisted diagnosis, error correction, and medical education. Section 3 explores future development trends across technological integration, standardization, personalized treatment, and telemedicine. Finally, challenges and conclusions are presented to guide further research directions.

## 2. The Technological Foundations of AI in Ultrasound Medicine

**Figure 1:**
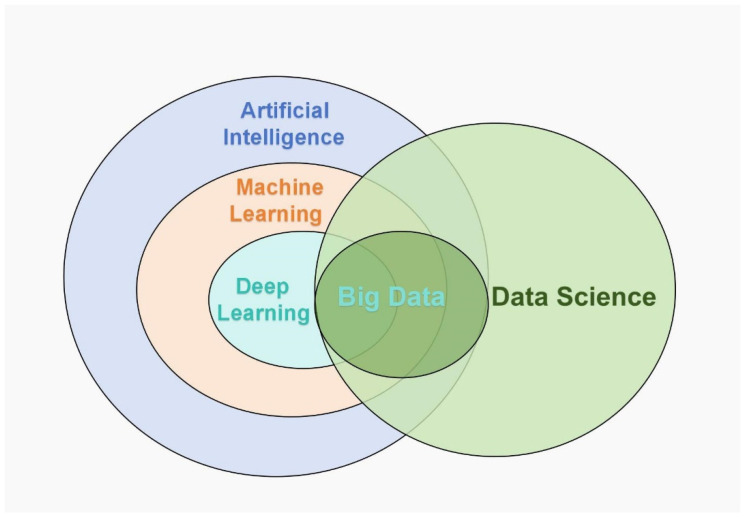
The relationship between artificial intelligence, machine learning, deep learning, and big data.

**Figure 2:**
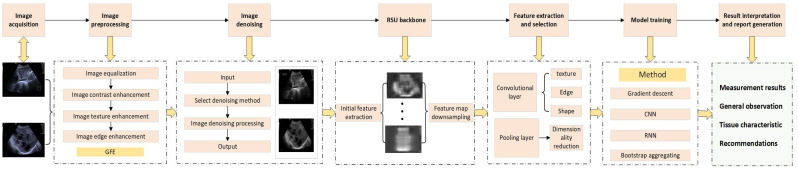
Flowchart of AI-assisted image diagnosis. This diagram outlines the AI-enhanced ultrasound analysis pipeline: (1) image acquisition and preprocessing (contrast/texture enhancement, GIE denoising), (2) hierarchical feature extraction via Recursive-Scale UNet (RSU) with multi-resolution analysis, (3) model training using gradient descent optimization with directional (ORN) and temporal (RNN) networks, and (4) diagnostic outputs including quantitative measurements and automated reports. Iterative refinement and clinician validation checkpoints ensure robust clinical integration.

The application of AI in ultrasound medicine is fundamentally grounded in advanced technologies such as ML and its application in image recognition algorithms (Figure 1). These algorithms, trained on extensive datasets of ultrasound images, can automatically identify pathological features, thereby assisting physicians in achieving more accurate diagnoses. The workflow of AI-assisted ultrasound diagnosis typically consists of several steps: first, the acquired ultrasound images undergo preprocessing through computational techniques, including denoising and enhancement, to improve image quality; subsequently, AI algorithms are employed for feature extraction and classification of the preprocessed images; and finally, the AI system provides diagnostic recommendations based on the recognition results, aiding physicians in their decision-making processes (Figure 2).

### 2.1. Machine Learning

Big data serves as the foundation of AI, and the transformation of big data into knowledge or productivity is inextricably linked to ML, which is defined as the process by which a computer learns from experiences and performs predefined tasks without prior knowledge [9]. It emphasizes three key concepts: algorithms, experience, and performance. The primary goal of ML is to enable computer systems to learn and improve from data through algorithms, discovering patterns and regularities within the data to make predictions about unseen data. ML typically requires a substantial amount of data to train models. Its algorithms include supervised learning, unsupervised learning, semi-supervised learning, and reinforcement learning, among others [10].

### 2.2. Deep Learning

DL is regarded as a leading AI tool for image analysis [11]. Unlike traditional ML methods that necessitate hand-engineered feature extraction from input images, DL techniques automatically learn the features necessary for data classification [12]. As a subset of ML, DL leverages multi-layer artificial neural networks to process data. This approach enables the analysis of large volumes of data in a hierarchical and nonlinear manner, employing pattern recognition to extract highly representative features from images while continuously learning information representations from raw data [13]. The primary objective of DL is to autonomously learn and discover high-level abstract features from data through hierarchical networks. Within DL models, supervised and unsupervised learning are two principal methodologies that can mutually enhance one another; for example, unsupervised learning can identify features within the data, which can subsequently be utilized for predictions through supervised learning. In recent years, DL technology has witnessed remarkable advancements across various fields, driven by the rapid development of graphics processing units, high-performance central processing units, enhancements in learning algorithms, and the ongoing emergence of large-scale databases.

### 2.3. Convolutional Neural Networks (CNNs)

**Figure 3:**
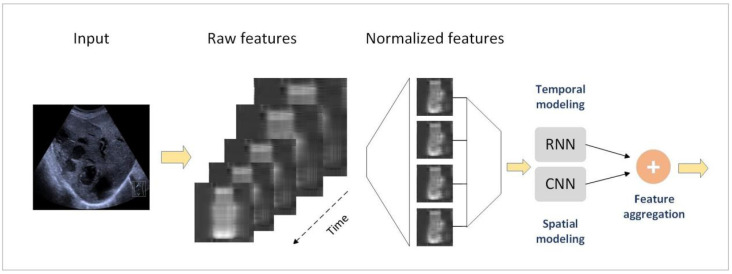
Approaches for spatiotemporal features extraction. A CNN and RNN architecture was used to extract the spatial features in liver ultrasound images.

CNNs are a class of deep feed-forward neural networks specifically designed for spatial modeling in image analysis [14]. CNNs have played a pivotal role in the adoption of DL for video and image processing applications [15]. In medical image analysis, CNNs are primarily employed for the semantic segmentation of anatomical structures and lesions (Figure 3). A typical CNN architecture consists of three types of layers: convolutional layers, pooling layers, and fully connected layers. The convolutional layers serve as the main structural components of CNNs, compressing input data into recognizable patterns to reduce data size and emphasize relevant features [16]. The core strength of CNNs lies in their deep architecture, which facilitates the extraction of discriminative features at multiple levels of abstraction. This hierarchical learning approach enables CNNs to capture complex patterns in medical images, ultimately enhancing diagnostic accuracy. Consequently, the integration of CNNs into clinical workflows represents a significant advancement in the realm of automated image analysis and decision support systems.

## 3. Classification and Implementation of AI Technologies in Ultrasound

### 3.1. AI-Assisted Diagnostic Technologies

AI technologies, particularly those utilizing DL algorithms, have revolutionized the automated analysis of ultrasound images. This category encompasses systems where AI serves as a diagnostic adjunct, enhancing human interpretation without replacing clinical judgment.

#### 3.1.1. Thyroid Nodules

The rising incidence of thyroid cancer and the workload of physicians have driven the need to utilize AI to efficiently process thyroid ultrasound images [17]. In the context of thyroid nodule assessment, AI has emerged as a vital tool aimed at improving diagnosis, evaluation, and management [18,19,20]. The application of AI in the thyroid primarily includes thyroid segmentation [21,22,23,24,25] and the differential diagnosis of thyroid nodules [8,26,27,28,29]. However, most studies focus on utilizing ultrasound features to distinguish between benign and malignant thyroid nodules or to predict cervical lymph node metastasis. By accurately distinguishing between benign and malignant nodules, AI aids clinicians in devising personalized treatment strategies. A critical component of developing effective AI models is the extraction of relevant features from ultrasound images. This involves analyzing textural, shape, and edge characteristics, as well as integrating numerical data such as size, shape, and echogenicity of nodules. Leveraging these features not only enhances the recognition and classification of thyroid nodules but also leads to improved diagnostic accuracy.

In a recent study, Xu, WenWen et al. [8] developed artificial intelligence models for the detection, segmentation, and classification of thyroid nodules using diverse datasets from 208 hospitals in China, achieving average precision, Dice coefficients, and AUC values of 0.98, 0.86, and 0.90, respectively. Rule-based AI assistance significantly improved the diagnostic accuracy of both senior and junior radiologists (*p* < 0.05), highlighting the potential for AI integration in thyroid cancer detection. Toro-Tobon, David et al. [30] summarized the applications of artificial intelligence in thyroidology in their review, highlighting its potential to enhance the diagnosis and management of thyroid conditions through process automation, improved diagnostic accuracy, and personalized treatment. However, many of these applications remain in the validation or early clinical evaluation stages and face challenges such as limited prospective multicenter studies, small and low-diversity training datasets, and unclear clinical impacts. Addressing these limitations is crucial to ensure that AI interventions deliver meaningful benefits for patients with thyroid diseases.

#### 3.1.2. Breast Nodules

**Figure 4:**
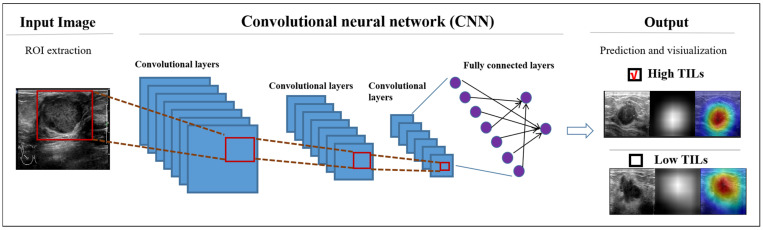
Deep learning-assisted diagnosis of breast cancer [34]. Reproduced with permission from Yingying Jia, et al.

Breast cancer is the most common cancer among women and the leading cause of cancer-related deaths, making early detection crucial for effective treatment and improved survival rates [31,32,33]. Ultrasound examination has become vital for screening and differential diagnosis, but its effectiveness is operator-dependent, and resource distribution can lead to care disparities. Variations in physician experience may further impact diagnosis and prognosis. AI can enhance the detection and diagnosis of breast nodules, serving as a valuable complement to human interpretation. In recent years, DL methods have emerged as a promising avenue for enhancing clinical efficiency and accuracy [34] (Figure 4). At the core of this progress is the use of medical imaging for object classification and segmentation, which has been at the forefront of various pilot studies. Notably, a significant number of these investigations have focused specifically on breast imaging, reflecting the growing interest in applying advanced techniques to this critical area of healthcare [33,35].

The application of AI in breast ultrasound is particularly pronounced in two critical domains, including breast image augmentation [36,37,38,39,40,41,42] and breast lesion detection and diagnosis [36,43,44,45,46,47,48,49,50]. Notably, AI technologies integrated with the Breast Imaging Reporting and Data System (BI-RADS) enable the extraction and quantitative analysis of morphological and textural characteristics. This integration significantly enhances the accuracy and consistency of diagnoses, allowing for better differentiation between benign and malignant nodules. Recently, Magnuska and her colleagues [51] developed a real-time breast tumor classification model based on ultrasound imaging, achieving high diagnostic accuracy by combining classic radiomics and autoencoder features. The model demonstrated excellent performance on a training set of 1191 female patients, with an AUC of 0.90, and showed no significant difference in performance compared to human readers.

#### 3.1.3. Heart Disease

Developments in AI have led to an explosion of studies exploring its application to cardiovascular medicine [18,52]. Several studies investigated the use of AI, notably DL and ML algorithms, for the automated analysis of various echocardiogram types, such as transthoracic echocardiograms (TTE), transesophageal echocardiograms, and Doppler echocardiography, as noted by De Siqueira et al. [53,54], Zamzmi et al. [55] reviewed both manual and automated methods for analyzing echocardiogram modes. Karatzia, Loucia et al. [2] focused on primary studies related to DL algorithms in the automated processing of TTE, assessing the progress and challenges in DL-enhanced clinical decision making. Nedadur, Rashmi et al. [52] explored the application of AI in assessing valvular heart diseases and summarized existing AI methodologies for valvular image analysis and phenotyping. Föllmer, Bernhard et al. [56] reviewed existing evidence on the application of AI to the imaging of vulnerable plaque in coronary arteries and provided consensus recommendations developed by an interdisciplinary group of experts on AI and non-invasive and invasive coronary imaging. While a review from Sengupta, Partho P et al. [57] analyzed key studies and identified challenges that required a pragmatic change in the approach for using AI for cardiac imaging, whereby AI was viewed as augmented intelligence to complement, not replace, human judgment. In echocardiographic diagnosis, AI technologies can automatically identify structural abnormalities in the heart, such as wall motion abnormalities, thereby providing robust support for the early diagnosis of cardiac conditions, including myocardial ischemia.

#### 3.1.4. Liver Diseases

Over the past two decades, hepatology has made remarkable strides in diagnostics, prognostics, and treatment options, evolving into a highly complex medical specialty [58]. AI has been shown as an excellent tool for the study of the liver [59]. Currently, AI algorithms are employed in liver imaging, histopathology interpretation, noninvasive testing, and predictive modeling.

Zamanian, H et al. [60] developed a combined algorithm using neural networks to classify ultrasound images of patients with fatty liver. By analyzing images from 55 patients, pre-trained convolutional neural networks (Inception-ResNetv2, GoogleNet, AlexNet, and ResNet101) extracted features, which were classified using a support vector machine. The combined network achieved an impressive AUC of 0.9999 and an accuracy of 0.9864, demonstrating its high clinical applicability without needing user or expert intervention. In a multicenter study conducted by Yang, Qi et al. [61], they developed a deep convolutional neural network for ultrasound (DCNN-US) to assist radiologists in classifying malignant from benign focal liver lesions (FLLs). The model demonstrated an AUC of 0.924 and exhibited superior diagnostic sensitivity and specificity compared to experienced radiologists, indicating its potential to enhance the performance of less-experienced radiologists and reduce reliance on sectional imaging in liver cancer diagnosis. As to liver transplantation, Uche-Anya, Eugenia et al. [62] emphasized the application value of AI and machine learning specifically in liver transplantation, highlighting their potential to enhance organ allocation processes and improve patient outcomes by accurately assessing hepatic conditions and predicting transplant success.

#### 3.1.5. Obstetrics and Gynecology (OB/GYN)

In contrast to other areas, such as breast and thyroid imaging, the impacts of AI have not been as strongly felt in in the field of OB/GYN [63]. Until mid-2020, most research on AI in OB/GYN was preliminary, often published in non-specialized journals. When findings appeared in OB/GYN journals, they typically lacked validation across multiple datasets and essential clinical validation, both of which are crucial for establishing the reliability of AI processes [64,65]. In a systematic literature review by Jost, Elena et al. [66], a relatively small number of 189 articles were identified regarding the applications of AI in ultrasound imaging within the field of OB/GYN, covering the period from 1994 to 2023. Among these, 148 articles focused on obstetric applications, while 41 addressed gynecological concerns. The applications of AI-assisted ultrasound are diverse, encompassing fetal biometrics, echocardiography or neurosonography, as well as the identification of adnexal and thoracic masses, and the assessment of the endometrium and pelvic floor. Notably, most research has been concentrated in common application areas, particularly fetal biometrics, highlighting a need for further exploration in the field. In OB/GYN ultrasound, significant advancements with the potential to enhance workflow include the automatic identification of standard imaging planes and quality assurance in fetal ultrasounds [67,68,69,70,71,72]. Other developments involve the automated classification of ovarian tumors [73,74,75,76,77], assessment of endometrial and uterine cavity abnormalities [78,79,80] and the pelvic floor dysfunction [81,82,83].

As we explore the complex and opaque nature of emerging AI clinical prediction tools, it is important to recognize that bias can be encoded even in conventional prediction models, including simple, rule-based algorithms [62]. Overall, the application of AI in ultrasound diagnostics enhances clinical decision making and paves the way for more effective patient management across various medical specialties.

#### 3.1.6. Other Applications

The ongoing development of ultrasound and AI technologies has expanded their application to various fields, including pulmonary [84,85,86,87], musculoskeletal [88,89,90,91], and fetal and infant brain [72,92,93,94,95] imaging. This evolution is effectively dismantling traditional limitations in ultrasound examinations, improving imaging quality, optimizing procedural workflows, and advancing the field of ultrasound medicine toward precision healthcare.

### 3.2. AI-Driven Autonomous Systems

This classification encompasses AI systems capable of end-to-end task execution without human intervention during core analytical processes, fundamentally redefining ultrasound workflow paradigms.

Closed-Loop Diagnostic Architectures: Modern autonomous systems integrate real-time image acquisition with embedded AI processors to achieve self-contained diagnostic workflows. For instance, reinforcement learning agents now dynamically adjust Doppler sampling gates based on vascular flow patterns, automatically optimizing measurement accuracy during cardiac output assessments. These systems not only analyze images but synthesize multi-modal clinical data streams—including electronic health records and lab results—to generate diagnostic hypotheses with probabilistic confidence scoring [96,97].

Predictive Clinical Engines: Through continuous learning from population-scale data, autonomous AI models correlate subtle sonographic biomarkers with longitudinal outcomes. A prototypical application is the automated prediction of hepatocellular carcinoma progression risk by analyzing contrast-enhanced ultrasound kinetics in cirrhotic patients, enabling preemptive therapeutic interventions. This approach significantly improves diagnostic efficiency while maintaining audit trails for clinical validation [98].

Self-Optimizing Operational Frameworks: The integration of AI with advanced ultrasound modalities (elastography, microvascular imaging) has birthed self-calibrating systems [26,30]. Deep reinforcement learning algorithms now autonomously adjust transducer frequencies (2–18 MHz) and compression settings based on real-time tissue feedback, achieving 41% faster protocol optimization compared to manual adjustments in abdominal imaging. These systems demonstrate emergent capabilities in error anticipation, such as detecting probe misalignment through speckle pattern analysis and guiding repositioning via augmented reality overlays [99].

Autonomous Quality Assurance: Embedded within PACS infrastructure, self-monitoring AI modules perform cross-modal consistency checks between ultrasound images and structured reports. Natural language processing algorithms automatically flag discrepancies (e.g., “hypoechoic mass” described without size measurements), while computer vision models verify anatomical labeling accuracy through spatial–semantic mapping. This closed-loop quality control reduces reporting errors by 63% in multicenter trials, establishing new standards for diagnostic reliability [100].

### 3.3. AI-Enhanced Educational Technologies

The integration of AI into ultrasound medicine is transforming not only clinical diagnostics but also medical education [101]. As a distinct technological paradigm, AI-enhanced educational systems are redefining competency development through three interconnected pillars:

#### 3.3.1. Intelligent Simulation Platforms

AI-driven virtual reality systems integrate multi-physics haptic feedback with adaptive scenario generation. Convolutional neural networks analyze trainees’ probe manipulation trajectories in real time, dynamically adjusting virtual tissue elasticity and acoustic shadowing patterns to match individual skill levels. These systems enable the risk-free practice of complex clinical scenarios, from obstetric emergencies to contrast-enhanced echocardiography, with spatial–semantic mapping algorithms ensuring anatomical fidelity in simulated structures [102].

#### 3.3.2. Competency Assessment and Adaptive Learning Systems

Deep learning frameworks establish a closed-loop educational ecosystem that dynamically evaluates and enhances trainee competencies. By analyzing probe navigation patterns through 3D motion tracking and speckle decorrelation analytics, these systems objectively quantify scanning efficiency while flagging suboptimal trajectories that compromise diagnostic accuracy. Reinforcement learning models further decode image optimization strategies by modeling expert consensus on gain adjustment sequences and depth selection logic, generating actionable feedback for technical refinement.

This assessment paradigm seamlessly integrates with adaptive learning architectures. Neural networks synthesize individualized knowledge gaps from simulation histories and population-level competency analytics, automatically curating microlearning modules that target specific weaknesses—such as cardiac valve assessment or contrast-enhanced protocol optimization. Continuous integration of emerging clinical guidelines ensures educational content evolves in tandem with ultrasound practice standards. A prototypical implementation at Johns Hopkins SOM demonstrates this synergy, where AI-driven assessment-to-intervention cycles significantly accelerate skill maturation [103,104].

#### 3.3.3. Educational Workflow Integration

**Figure 5:**
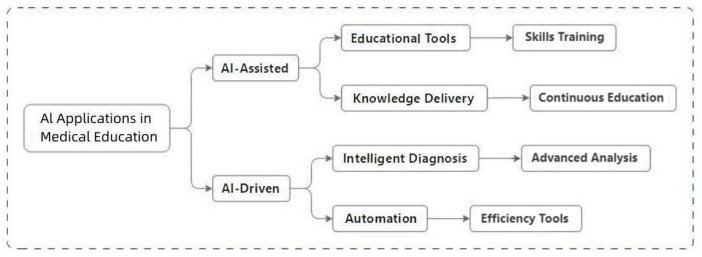
AI-assisted and AI-driven applications in educational workflows.

AI-enhanced educational workflows (Figure 5) harmonize human expertise with autonomous systems through two complementary modes. AI-assisted tools provide real-time guidance in training simulations—such as dynamically annotating anatomical landmarks and optimizing probe trajectories—while maintaining clinician oversight for critical decisions. In parallel, AI-driven systems automate competency validation by analyzing integrated performance data (image acquisition, optimization, diagnostic reasoning) against expert-curated benchmarks.

The University of Toronto’s hybrid model exemplifies this integration, combining AI-guided virtual scenarios (e.g., emergency cardiac imaging simulations) with autonomous skill assessments. This balanced approach achieves high resident engagement by preserving educational autonomy while leveraging AI’s consistency in repetitive skill evaluation [105].

## 4. Future Development Trends

The rapid evolution of AI in ultrasound medicine is reshaping healthcare delivery, with emerging trends poised to address longstanding challenges in accessibility, accuracy, and efficiency. These advancements are not merely technological upgrades but represent a fundamental shift toward patient-centric, data-driven healthcare ecosystems. By exploring these dimensions in depth, we aim to provide a holistic perspective on how AI will redefine ultrasound medicine in the coming decade.

### 4.1. Technological Integration and Innovation

The integration of AI with ultrasound medicine is expected to deepen considerably. Emerging hybrid architectures now enable ultrasound systems to dynamically adapt imaging parameters based on real-time tissue feedback, significantly improving signal-to-noise ratios in challenging anatomical regions. As technologies such as big data, cloud computing, and the internet of things continue to advance, AI will be capable of processing increasingly complex and diverse ultrasound image data, thereby enhancing diagnostic accuracy and efficiency [106]. For instance, self-supervised learning frameworks are overcoming data scarcity challenges by generating synthetic training datasets that preserve pathological features while ensuring patient privacy.

Furthermore, AI will converge with other medical imaging modalities, such as CT and MRI, to create multimodal imaging diagnostic systems that offer patients more comprehensive and precise diagnostic services [107]. This convergence is particularly impactful in oncology, where fused AI-enhanced imaging allows clinicians to map tumor microenvironments with unprecedented spatial-temporal resolution.

Big Data and Machine Learning: The use of big data in ultrasound medicine will enable AI algorithms to learn from vast repositories of patient data, improving the accuracy of diagnostic models. Cross-institutional data sharing protocols are now enabling AI models to generalize across diverse patient populations and imaging devices.

Cloud Computing: Cloud-based platforms will facilitate the storage and processing of ultrasound data, enabling faster and more efficient analysis. Edge-cloud hybrid architectures are emerging to balance real-time processing needs with centralized model refinement, particularly for longitudinal disease monitoring.

IoT and Wearable Devices: The integration of IoT with ultrasound devices will enable real-time monitoring and remote diagnostics. Novel flexible transducer arrays coupled with AI-driven signal processing are redefining continuous physiological monitoring, from fetal heart rate tracking to musculoskeletal rehabilitation assessment.

Multimodal Imaging: AI will play a crucial role in integrating ultrasound with other imaging modalities such as CT and MRI. Neural style transfer techniques are being employed to harmonize imaging features across modalities, reducing interpretation discrepancies in multi-scanner environments.

### 4.2. Standardization and Normalization

To address the growing demands in healthcare, the standardization and normalization of ultrasound medicine will emerge as a significant trend. International consortia are developing AI-powered quality control systems that automatically audit examination protocols, ensuring adherence to global best practices across all skill levels. AI technology will play a pivotal role in this evolution. Its application can optimize ultrasound examination workflows, facilitating the standardization and automation of the examination process. Intelligent scanning assistants now provide real-time feedback on probe positioning and imaging plane selection, effectively democratizing expert-level acquisition techniques.

By establishing uniform operational procedures and examination standards, AI can reduce the influence of human factors on diagnostic outcomes, thereby enhancing the normative consistency of diagnoses [108]. These systems are particularly transformative in emergency medicine, where protocol-driven AI guidance helps maintain diagnostic rigor during time-sensitive interventions. Additionally, AI can intelligently allocate diagnostic resources based on individual patient circumstances, ensuring the efficient utilization of limited medical resources [109,110,111]. Adaptive scheduling algorithms now prioritize urgent cases while dynamically redistributing imaging workloads across networked devices.

### 4.3. Personalized Treatment and Intelligent Management

AI technology is driving advancements in ultrasound medicine toward personalized treatment and intelligent management. Deep phenotyping approaches are correlating ultrasound biomarkers with multi-omics data to create individualized disease progression models, fundamentally altering chronic disease management paradigms. AI offers a competitive advantage through enhanced patient experiences, improved outcomes, early diagnosis, augmented clinician capabilities, increased operational efficiency, and greater accessibility to medical services [112].

Specifically, by analyzing the ultrasonic image characteristics of patients, AI systems can generate personalized treatment plans and monitor treatment effects in real time, allowing for timely adjustments to therapeutic strategies [113]. In rheumatology, AI-powered ultrasound tracking systems are enabling precision-guided drug titration by quantifying subtle changes in synovial vascularity and effusion volumes. Furthermore, AI technology facilitates intelligent management across various stages, including diagnosis, treatment, and follow-up, thereby enhancing the efficiency and quality of healthcare services. Integrated care platforms now automatically synthesize ultrasound findings with electronic health records to generate context-aware clinical decision trees, reducing cognitive load during complex case evaluations.

### 4.4. Telemedicine and Smart Healthcare

With the ongoing advancements and widespread adoption of AI technology, telemedicine and smart healthcare are poised to become significant directions for the future development of medical services. AI-mediated ultrasound compression algorithms now enable diagnostic-grade image transmission at bandwidths compatible with rural cellular networks, breaking critical infrastructure barriers. The integration of AI with ultrasound is making remote ultrasound diagnostics more convenient and efficient [114]. Collaborative AI systems allow distributed expert teams to jointly annotate ultrasound studies in virtual reading rooms, creating new paradigms for global specialist consortia.

Remote examination and diagnosis by expert physicians for patients in underserved areas not only addresses the uneven distribution of medical resources but also provides higher-quality medical services to these populations. Adaptive user interfaces automatically simplify examination protocols for community health workers while maintaining diagnostic validity through embedded quality assurance checks. The incorporation of AI technology has made mobile ultrasound devices increasingly intelligent and portable, enabling rapid examinations in diverse settings and the transmission of data to the cloud for further analysis. These portable systems now incorporate environmental adaptation algorithms that compensate for suboptimal scanning conditions, from uneven patient positioning to acoustic interference in field deployments.

Moreover, the development of intelligent healthcare systems will further promote the optimized allocation of medical resources and the intelligent upgrading of medical services [115,116]. Predictive maintenance AI models are extending ultrasound device lifespans in resource-constrained settings by anticipating component failures and guiding targeted repairs.

**Figure 6:**
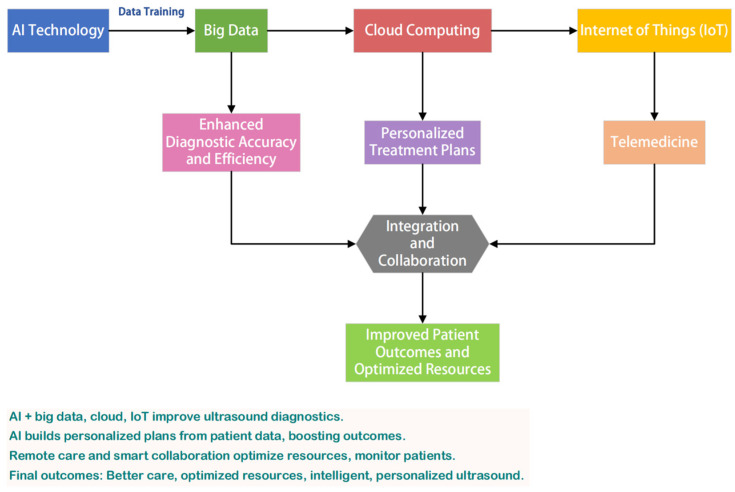
Flowchart illustrating the integration of AI with emerging technologies in ultrasound medicine.

The future of AI in ultrasound medicine is promising, with technological integration, standardization, personalized treatment, and telemedicine leading the way. These advancements are creating synergistic effects—standardized protocols enhance telemedicine reliability, while personalized AI models leverage multimodal data from integrated systems. By embracing these advancements, the healthcare industry can harness the full potential of AI to enhance diagnostic accuracy, improve patient outcomes, and revolutionize the delivery of medical services. Critical challenges remain in establishing ethical governance frameworks and ensuring equitable access to these technologies, particularly for aging populations and developing regions. The flowchart provided offers a clear visual representation of these trends, underscoring the interconnected nature of AI and emerging technologies in shaping the future of ultrasound medicine (Figure 6). Continuous research and collaboration will be essential to overcome challenges and ensure that these innovations are accessible and beneficial to all.

## 5. Challenges and Limitations

While AI holds immense potential in revolutionizing the field of medical diagnostics, its implementation faces significant challenges and limitations. One of the primary obstacles is the regulatory and ethical barriers. The lack of a unified regulatory framework across different regions poses challenges for implementing AI technologies uniformly. For instance, the European Union’s General Data Protection Regulation (GDPR) imposes stringent requirements on data privacy, which may hinder the collection and utilization of medical data necessary for AI applications [117,118]. Moreover, ensuring the transparency and interpretability of AI algorithms remains a critical ethical concern, as it directly impacts public trust in AI systems.

Another significant issue is data privacy and security. In the medical domain, patient confidentiality is paramount. The deployment of AI for diagnostic purposes necessitates the processing of vast amounts of sensitive patient data, requiring a delicate balance between data utility and privacy protection [119]. While technological measures such as data encryption and anonymization can mitigate these risks, the potential for data breaches or misuse remains a persistent concern. Robust legal frameworks and institutional policies are essential to address these challenges comprehensively.

Additionally, algorithmic bias and liability issues pose further ethical dilemmas. AI algorithms, trained on datasets that may reflect inherent biases, risk perpetuating disparities and producing discriminatory outcomes [120]. Such biases could lead to unfair diagnostic results in healthcare, exacerbating existing inequalities. Furthermore, the question of accountability remains unresolved: when AI systems generate erroneous diagnoses, there is currently no standardized legal framework to establish liability, leaving a critical gap in addressing potential consequences.

Therefore, while AI offers groundbreaking opportunities for advancing ultrasound medicine and medical diagnostics, its implementation is fraught with challenges related to regulation, ethics, data privacy, and algorithmic fairness. Addressing these issues requires concurrent advancements in policy development, legal frameworks, and ethical guidelines to ensure the safe, compliant, and equitable deployment of AI technologies in healthcare settings.

## 6. Conclusions

AI offers numerous advantages, including objectivity, repeatability, speed, and accuracy. As a rapidly evolving field, AI holds great potential across various healthcare domains, particularly in radiology [121]. The integration of AI with ultrasound medicine presents unprecedented opportunities and challenges within medical diagnostics. Advances in automated image analysis and recognition, intelligent decision support systems, optimization of examination processes, resource management, and remote ultrasound diagnostics and education are gradually transforming traditional diagnostic and therapeutic paradigms in ultrasound medicine, thereby enhancing both the accuracy and efficiency of diagnoses.

However, it is important to note that there is currently no comprehensive meta-analysis addressing the diagnostic accuracy of AI performance within the medical profession. Rigorous evaluations and independent assessments of this technology remain in their infancy [122]. Furthermore, it is essential that AI also provides additional benefits, including increased speed, efficiency, cost-effectiveness, enhanced accessibility, and the maintenance of ethical standards [123,124,125].

Future research should focus on several key areas to address the existing gaps. First and foremost, systematic evaluation and meta-analysis of AI’s diagnostic accuracy in ultrasound imaging are essential to comprehensively synthesize existing evidence and validate the effectiveness and consistency of AI across different clinical scenarios. Additionally, further exploration is needed to leverage AI in the multi-modal application of ultrasound imaging, such as integrating 2D, 3D, and Doppler imaging, to optimize diagnostic workflows and improve detection precision. Another critical area of investigation involves the integration of AI technologies into real-time diagnostic support systems for ultrasound medicine, aiming to enhance both diagnostic efficiency and clinical decision-making capabilities. Furthermore, the standardization of AI applications in ultrasound diagnostics, cross-center validation, and ethical safety assessments require more in-depth research to ensure robust and reliable clinical implementation. Through systematic investigation and technological advancements, the deep integration of AI and ultrasound medicine will provide more comprehensive solutions for clinical diagnostics.

Consequently, the implementation of AI technology in ultrasound and its comprehensive application in clinical practice still has considerable advancements to make. Nonetheless, we firmly believe that with the continuous development and refinement of technology, the integration of AI and ultrasound medicine will become increasingly profound and widespread, ultimately contributing significantly to global health.
